# Health Literacy and Acceptance of AI/XR-Enabled Telemedicine Among Romanian Medical Students: A Cross-Sectional Survey

**DOI:** 10.3390/healthcare14050570

**Published:** 2026-02-25

**Authors:** Codrina Mihaela Levai, Laura Alexandra Nussbaum, Adriana Cojocaru, Daian-Ionel Popa, Andrei Marius Tomescu, Iulius Jugănaru

**Affiliations:** 1 Doctoral School, “Victor Babes” University of Medicine and Pharmacy, 300041 Timisoara, Romania; codrinalevai@umft.ro (C.M.L.); daian-ionel.popa@umft.ro (D.-I.P.); andrei.tomescu@umft.ro (A.M.T.); 2 Research Center for Medical Communication, “Victor Babes” University of Medicine and Pharmacy of Timisoara, 300041 Timisoara, Romania; 3 Department of Neurosciences, “Victor Babes” University of Medicine and Pharmacy, 300041 Timisoara, Romania; adriana.cojocaru@umft.ro; 4 Department XI Pediatrics, Discipline I Pediatrics, “Victor Babes” University of Medicine and Pharmacy of Timisoara, 300041 Timisoara, Romania; juganaru.iulius@umft.ro; 5 Department of Research Center for Disturbances of Growth and Development in Children–BELIVE, “Victor Babes” University of Medicine and Pharmacy of Timisoara, 300011 Timisoara, Romania

**Keywords:** telemedicine, students, medical, artificial intelligence, virtual reality, health literacy

## Abstract

**Background and Objectives**: AI- and extended reality (XR)-enabled telemedicine is increasingly relevant to clinical training, yet evidence from Central and Eastern Europe is limited. We assessed Romanian medical students’ acceptance of AI/XR-enabled telemedicine and examined whether health literacy moderates the association between AI/XR knowledge and acceptance. **Methods**: We conducted an anonymous cross-sectional online survey of 212 medical students (years 1–6) at a single Romanian university (March 2024–June 2025). Acceptance was measured using a study-specific Acceptance Index (mean of three 4-point items: trust in AI-assisted recommendations, perceived improvement in telemedicine quality with AI/XR, and willingness to participate in AI/XR-enabled teleconsultations; internal consistency acceptable, Cronbach’s α ≈ 0.8). Health literacy was assessed with the validated Romanian version of the European Health Literacy Survey Questionnaire (HLS-EU-Q16). We performed group comparisons, Spearman correlations, multivariable and hierarchical regression with a Knowledge × Health Literacy interaction, and k-means clustering. **Results**: Participants had a mean age of 22.5 ± 1.9 years; 66.0% were female. Overall acceptance was high (2.9 ± 0.6). Acceptance was higher in clinical vs. preclinical years (3.1 ± 0.6 vs. 2.8 ± 0.5; *p* < 0.001; Cohen’s d ≈ 0.55) and in prior AI/XR users vs. non-users (3.2 ± 0.5 vs. 2.7 ± 0.6; *p* < 0.001; d ≈ 0.89). Knowledge correlated strongly with acceptance (ρ = 0.68; *p* < 0.001). In multivariable models (R^2^ = 0.61), knowledge, perceived educational value, prior AI/XR use, and clinical stage independently predicted acceptance, whereas privacy concern and gender did not. Health literacy was sufficient in 64.2% and significantly moderated the knowledge–acceptance link (interaction *p* = 0.012). **Conclusions**: Romanian medical students report favorable acceptance of AI/XR-enabled telemedicine. Findings support curriculum integration that combines structured AI/XR teaching with literacy-sensitive scaffolding to ensure knowledge translates into informed, critical acceptance across student subgroups.

## 1. Introduction

The rapid expansion of telemedicine after the COVID-19 pandemic has been accompanied by a parallel rise in digital augmentation tools, including artificial intelligence (AI) and extended reality (XR). For future physicians, these technologies will shape not only how they deliver remote care but also how they learn clinical reasoning, communication, and procedural skills. Global analyses show that the pandemic triggered an unprecedented surge in virtual care, with telehealth becoming embedded in routine service delivery across many health systems, particularly where regulatory and reimbursement barriers were temporarily relaxed [1,2]. At the same time, observers of digital health warn that technological progress has outpaced cultural and organizational readiness, creating a widening “adoption gap” that is especially visible in education and workforce development [3]. The emergence of generative AI tools such as ChatGPT has further accelerated experimentation with AI in triage, documentation, and clinical decision support, while also raising questions about transparency, bias, and professional identity in medicine [4].

For medical students, telemedicine and AI are not abstract policy concepts but concrete tools they encounter in courses, clerkships, and informal learning. Early survey work in China and other settings suggested that medical students were more familiar with telehealth than patients but often had limited hands-on experience and substantial concerns about data quality and reliability [5]. A recent systematic review of cross-sectional studies concluded that students’ attitudes toward telemedicine are generally positive, yet knowledge of specific applications and regulatory frameworks remains patchy, largely because dedicated teaching is rare in undergraduate curricula [6]. Country-level surveys, including work from the United States, report high perceived usefulness of teleconsultations among students but inconsistent preparedness to conduct them independently [7]. In parallel, studies of eHealth learning show that most medical students welcome digital health content and explicitly ask for structured training in these domains, rather than encountering telemedicine and digital tools only informally during rotations [8].

Acceptance among trainees is not a trivial attitudinal detail; it is a prerequisite for meaningful adoption. If students regard AI-enhanced teleconsultations as opaque, risky, or pedagogically unhelpful, they may under-utilize tools that future health systems will expect them to master. Conversely, over-enthusiastic, uncritical acceptance could create blind spots around safety, bias, and equity. In Romania, a recent scoping review of telemedicine education highlighted fragmented, uneven exposure to digital health topics across universities, with substantial variability in learning objectives, teaching methods, and assessment strategies [9]. Broader analyses of the digital transformation of Romanian health services likewise document uneven infrastructure, regional disparities in connectivity, and a mismatch between patients’ expectations and the system’s capacity to deliver integrated, digitally supported care [10]. In such a context, understanding where students currently sit on the spectrum between skepticism and hype is essential for designing curricula that are both engaging and ethically grounded.

AI and XR offer distinct but complementary affordances in telemedicine. AI can triage symptoms, flag abnormal imaging, or suggest management options during virtual visits, while XR can recreate examination rooms, procedural fields, or rehabilitation environments through immersive headsets. Recent multinational surveys of medical students and trainees report high awareness of AI in medicine and widespread expectations that it will improve diagnostic accuracy and efficiency, but also concerns about job displacement, professional autonomy, and the impact on patient–physician relationships [11,12]. When AI is used explicitly as an educational tool—such as AI-supported question banks or tutoring systems—students often describe it as effective and time-saving, yet express ambivalence about credibility and the risk of superficial learning [13]. For students participating in or observing teleconsultations, AI and XR could theoretically improve feedback, realism, and autonomy, but they also introduce worries about over-reliance on algorithms, depersonalization of care, and the handling of sensitive patient data.

Evidence for XR-based interventions in health professions education has expanded rapidly. Systematic reviews and meta-analyses suggest that virtual reality–based training can improve knowledge, skills, and learner engagement compared with traditional methods, particularly for procedural and spatially complex tasks [14]. However, most existing XR studies focus on simulation laboratories or operating room scenarios rather than on telemedicine-like encounters, and few explicitly examine how immersive environments interact with AI-driven feedback or clinical decision support. At the same time, recent work on the “digital readiness” of future physicians underscores that comfort with digital tools is uneven across specialties, countries, and stages of training, and that curricular reforms often lag behind students’ lived experience with technology in their personal lives [15].

Health literacy is conceptually related to, but not synonymous with, digital health literacy (or eHealth literacy) and emerging constructs such as AI literacy. While digital health literacy emphasizes skills for navigating digital sources and tools, general health literacy reflects broader competencies in accessing, understanding, appraising, and applying health information across contexts. We focused on general health literacy because a validated Romanian version of the HLS-EU-Q16 is available and because these upstream competencies plausibly shape how students interpret, evaluate, and trust AI/XR-enabled telemedicine outputs [16]. We recognize that future studies should directly measure digital/AI-specific literacy and compare their explanatory value.

Despite rapid digitalization, evidence from Central and Eastern Europe remains limited, and Romanian medical education has been described as having fragmented and uneven exposure to telemedicine and related digital competencies. Beyond access to technology, adoption also depends on the learner’s capacity to find, understand, appraise, and apply health-related information in digital contexts. In educational telemedicine scenarios enhanced by AI/XR, these skills may influence how students interpret algorithm-supported recommendations, evaluate uncertainty and bias, and balance perceived educational value against privacy or safety concerns. We therefore focus on health literacy as a plausible upstream determinant that may shape how domain-specific knowledge translates into acceptance of AI/XR-enabled telemedicine

The present study therefore aimed to describe medical students’ acceptance of AI/XR-enabled telemedicine within a Romanian university setting, quantify perceived educational value and privacy concerns, and identify factors associated with higher acceptance. We hypothesized that acceptance would be higher among clinical-year students and those with prior AI/XR experience, and that knowledge and perceived educational value would show positive associations with acceptance, whereas privacy concerns would show little or no association.

## 2. Materials and Methods

### 2.1. Study Design, Setting, and Ethics

We conducted a cross-sectional survey at Victor Babes University of Medicine and Pharmacy Timisoara, Romania. The study targeted undergraduate medical students enrolled in the six-year general medicine program. Data collection took place between March 2024 and June 2025. The survey was administered online using a secure university-hosted platform. Participation was voluntary and anonymous. Before accessing the questionnaire, students read an information page describing the study purpose, data handling, and their rights, and indicated informed consent. No incentives were provided. The study protocol was reviewed and approved by the institutional ethics committee of Victor Babes University of Medicine and Pharmacy Timisoara, in alignment with national regulations and the Declaration of Helsinki.

### 2.2. Participants and Recruitment

All students registered in years 1–6 of the general medicine program were eligible if they were at least 18 years old and able to complete the survey in Romanian. There were no exclusion criteria related to prior exposure to telemedicine or AI/XR tools, as heterogeneity in experience was of interest. We distinguished between preclinical students (years 1–3) and clinical students (years 4–6), reflecting major curricular differences in patient contact and use of telemedicine platforms. This dichotomy reflects the program structure in which years 1–3 emphasize foundational sciences with limited patient contact, whereas years 4–6 involve clinical clerkships with greater exposure to real-world workflows, including telemedicine-related encounters.

Recruitment used a convenience strategy. The survey link and QR code were circulated via official year-group mailing lists, virtual learning environments, and student association channels. To reduce repeat participation, students were asked to submit the questionnaire only once, and the platform was configured not to store incomplete submissions. Although we could not calculate a precise response rate due to forwarding across informal channels, the final sample of 212 students represented all six years and both preclinical and clinical stages.

### 2.3. Survey Instrument and Measures

The 20-item questionnaire was developed by a multidisciplinary team (medical education, infectious diseases, and biomedical informatics) based on prior work on telemedicine acceptance and pilot AI/XR teaching activities in the institution. Items covered demographics (age, gender, study year), prior use of telemedicine in any role (as student or patient), prior exposure to AI or XR tools, and attitudinal constructs relevant to acceptance. Response options were Likert-type with 4 levels, harmonized so that higher scores generally represented more favorable views, except for privacy concern.

Key attitudinal variables included self-rated knowledge about AI/XR in healthcare and education (1 = not informed, 4 = very informed), perceived educational value of AI/XR-enabled telemedicine (1 = no added value, 4 = very high added value), privacy concern about data use in AI-assisted teleconsultations (1 = not concerned, 4 = very concerned), and willingness to invest additional personal time in training on AI/XR tools (1 = not willing, 4 = very willing). Acceptance was assessed using three items: trust in AI-assisted recommendations during teleconsultations, perceived improvement in telemedicine quality when AI/XR is present, and willingness to participate in AI/XR-enabled teleconsultations as part of training. The Acceptance Index was calculated as the mean of these items on a 1–4 scale; internal consistency was good (Cronbach’s α ≈ 0.8).

For analysis, we treated age as a continuous variable and grouped study year into preclinical (1–3) and clinical (4–6). Prior telemedicine use and prior AI/XR use were coded dichotomously (yes/no). The primary outcome was the continuous Acceptance Index. Secondary outcomes were each attitudinal item, particularly knowledge, perceived educational value, privacy concern, and willingness to invest extra training time.

Because recruitment was limited to a single institution and relied on convenience sampling, this study should be interpreted as exploratory and primarily hypothesis-generating with respect to broader national generalizability.

The AI/XR attitudinal items and the Acceptance Index were study-specific measures developed by adapting themes commonly used in technology acceptance research (e.g., perceived usefulness/value, trust, and willingness to engage). Prior to deployment, items were reviewed by the multidisciplinary author group for clarity and face validity, and the Acceptance Index was evaluated for internal consistency in the present sample; however, external psychometric validation was not performed and is noted as a limitation.

Privacy concern was not reverse-scored; higher values indicate greater concern. For regression models, coefficients are interpreted accordingly (negative coefficients would indicate lower acceptance at higher concern.

### 2.4. Health Literacy Questionnaire and Advanced Statistical Analyses

To complement the AI/XR-specific items, we administered the European Health Literacy Survey Questionnaire, short form (HLS-EU-Q16), Romanian version, a previously validated instrument for assessing general health literacy in the Romanian population [17]. The tool comprises 16 items covering the ability to access, understand, appraise, and apply health information in healthcare, disease prevention, and health promotion contexts. Each item is rated on a 4-point scale (very difficult, fairly difficult, fairly easy, very easy) and scored according to standard recommendations, yielding a summary index from 0 to 16, where higher scores indicate better health literacy. In line with the original cut-offs, students were categorized as having inadequate (0–8), problematic (9–12), or sufficient (13–16) health literacy. Internal consistency of the HLS-EU-Q16 in our sample was acceptable (Cronbach’s α ≈ 0.8), and the scale was treated as both categorical (for descriptive comparisons) and continuous (for regression modelling).

Acceptance Index items were constructed to reflect three core acceptance facets (trust, perceived quality improvement, willingness to participate) that are frequently used in technology-acceptance research; wording was adapted for the AI/XR telemedicine education context. Acceptance Index questionnaire is presented in Table 1.

Beyond the basic comparisons described above, the HLS-EU-Q16 index was incorporated into a set of advanced analyses designed to explore more nuanced relationships between health literacy and acceptance of AI/XR-enabled telemedicine. First, we extended the main linear regression on the Acceptance Index to a hierarchical model. Hierarchical blocks were specified a priori to reflect plausibly ordered determinants: (Model 1) demographic/training stage and prior exposure; (Model 2) attitudinal determinants reflecting perceived benefit and feasibility; (Model 3) the literacy-by-knowledge interaction. Although perceived educational value and willingness-to-pay are conceptually related, they capture different constructs in this study (perceived pedagogic benefit versus willingness to accept additional personal cost/effort). Multicollinearity diagnostics were used to ensure stable estimation.

Model fit was assessed using R^2^ and incremental ΔR^2^, and we reported unstandardized regression coefficients with 95% confidence intervals.

To visualize and further probe the moderation effect, we conducted a simple-slope analysis. Students were split at the median HLS-EU-Q16 score into “low” and “high” health-literacy groups, and separate linear regressions were run in each stratum with the Acceptance Index as the dependent variable and knowledge as the predictor. The resulting slopes and their 95% confidence intervals were compared, and model R^2^ was reported for each subgroup to quantify how strongly knowledge explained acceptance at different literacy levels. Finally, to identify distinct patterns of co-occurring attitudes, we performed a k-means cluster analysis (k = 3) on z-standardized scores of the Acceptance Index, AI/XR knowledge, willingness to pay, and HLS-EU-Q16. The choice of three clusters (k = 3) was guided by inspection of the within-cluster sum of squares (elbow method) and silhouette diagnostics, as well as interpretability. Cluster profiles were then compared using one-way ANOVA (or Kruskal–Wallis tests where appropriate), with partial η^2^ reported as an effect-size index.

For the simple-slope visualization, we used a median-based split of the HLS-EU-Q16 to create similarly sized ‘lower’ and ‘higher’ literacy strata for interpretability; the primary moderation inference is based on the continuous interaction term in the hierarchical regression.

### 2.5. Statistical Analysis

Data were exported to a local statistical package and checked for completeness, outliers, and logical inconsistencies. Because the platform required all core items to be completed before submission, there were no missing values on primary variables. Continuous variables are summarized as means and standard deviations (SD), and categorical variables as counts and percentages. Normality was assessed using histograms and Shapiro–Wilk tests; attitudinal scores showed moderate skew, so non-parametric tests were preferred for group comparisons. With *n* = 212 and a limited number of predictors in multivariable models, the analysis maintained an adequate observations-to-predictor ratio for linear regression, supporting stable coefficient estimation; cluster analysis was treated as exploratory.

We compared preclinical and clinical students using independent-samples *t*-tests for age and Mann–Whitney U tests for ordinal scores. Differences in proportions (e.g., gender, prior telemedicine, and prior AI/XR use) were examined with chi-square tests. Associations between attitudinal variables were quantified with Spearman’s rank correlation coefficients (ρ). To identify independent predictors of Acceptance, we fit a multiple linear regression model with the Acceptance Index as the dependent variable and knowledge, perceived educational value, privacy concern, prior AI/XR use, study stage, and gender as predictors. Model fit was evaluated using R^2^, and coefficients were reported with 95% confidence intervals (CI). Two-sided *p*-values < 0.05 were considered statistically significant.

## 3. Results

Table 2 summarizes the demographic characteristics and prior exposure to telemedicine and AI/XR across the total sample of 212 students and by study stage. The overall mean age was 22.5 ± 1.9 years, with clinical students being substantially older than preclinical peers (24.1 ± 1.3 vs. 21.2 ± 1.2 years, *p* < 0.001). Females represented two-thirds of the sample (140/212, 66.0%), with a similar gender distribution in preclinical (68.3% female) and clinical years (63.0% female; *p* = 0.509). Prior telemedicine use was reported by 56.6% of participants (120/212), but this was significantly more common among clinical students (65.2%) than preclinical students (50.0%; *p* = 0.038), consistent with greater clinical exposure. In contrast, prior AI/XR use was reported by 38.7% of the sample (82/212) and did not differ significantly between preclinical (40.8%) and clinical students (35.9%; *p* = 0.553), suggesting that hands-on experience with AI/XR tools is still relatively limited and not strongly stage-dependent.

Table 3 presents AI/XR-related attitudinal scores by study stage, showing modest but consistent advantages for clinical students. Knowledge about AI/XR on a 1–4 scale was higher in clinical years (2.7 ± 0.8) than in preclinical years (2.3 ± 0.8; *p* < 0.001), and perceived educational value of AI/XR-enabled telemedicine was also slightly higher in the clinical group (3.2 ± 0.6 vs. 3.0 ± 0.6; *p* = 0.023). Privacy concern scores were mid-range overall (2.5 ± 0.7), with no statistically significant stage difference (2.6 ± 0.7 in clinical vs. 2.4 ± 0.6 in preclinical; *p* = 0.094). The Acceptance Index showed a clear gradient, with clinical students reporting higher acceptance (3.1 ± 0.6) compared with preclinical students (2.8 ± 0.5; *p* < 0.001). Willingness to invest extra training time was high across the sample (overall 3.7 ± 0.5), but clinical students again scored slightly higher (3.8 ± 0.4 vs. 3.7 ± 0.5; *p* = 0.013), suggesting that more advanced students may be particularly ready to engage with AI/XR-enhanced telemedicine teaching.

Table 4 compares AI/XR-related scores between students with and without prior AI/XR use, pooling preclinical and clinical years. Prior users (*n* = 82) showed markedly higher Acceptance Index scores than non-users (3.2 ± 0.5 vs. 2.7 ± 0.6; *p* < 0.001) and reported substantially greater knowledge about AI/XR (2.9 ± 0.7 vs. 2.1 ± 0.8; *p* < 0.001). Perceived educational value of AI/XR-enabled telemedicine was also higher among prior users (3.3 ± 0.6) compared with non-users (3.0 ± 0.6; *p* < 0.001). Privacy concern was slightly higher in the prior-use group (2.6 ± 0.7 vs. 2.4 ± 0.7), although this difference did not reach statistical significance (*p* = 0.084), suggesting that familiarity does not necessarily reduce concerns about data protection. Willingness to invest training time was near ceiling among prior users (3.9 ± 0.3) and still high among non-users (3.6 ± 0.6), with a significant difference between groups (*p* < 0.001), indicating that prior exposure to AI/XR is strongly associated with enthusiasm and readiness to invest in further learning.

Knowledge about AI/XR showed a strong positive correlation with the Acceptance Index (ρ = 0.68, *p* < 0.001) and moderate positive correlations with perceived educational value (ρ = 0.49, *p* < 0.001) and willingness to invest time (ρ = 0.42, *p* < 0.001). Acceptance itself correlated strongly with perceived value (ρ = 0.60, *p* < 0.001) and moderately with willingness to invest time (ρ = 0.36, *p* < 0.001). In contrast, privacy concerns were only weakly and non-significantly related to knowledge (ρ = 0.05, *p* = 0.463), acceptance (ρ = 0.05, *p* = 0.498), perceived value (ρ = 0.03, *p* = 0.621), and willingness to invest time (ρ = 0.03, *p* = 0.651), as seen in Table 5.

Table 6 presents the results of a multiple linear regression model predicting the Acceptance Index from knowledge, perceived educational value, privacy concerns, prior AI/XR use, study stage, and gender. The model explained 61% of the variance in acceptance (R^2^ = 0.61), indicating a substantial overall fit. Higher knowledge scores were associated with higher acceptance (β = 0.28, 95% CI: 0.20–0.36; *p* < 0.001), as were higher perceived educational value scores (β = 0.32, 95% CI: 0.23–0.41; *p* < 0.001). Prior AI/XR use remained a significant independent predictor (β = 0.25, 95% CI: 0.13–0.37; *p* < 0.001), as did being in the clinical stage versus preclinical (β = 0.18, 95% CI: 0.07–0.29; *p* = 0.001). Privacy concern showed a small, non-significant negative coefficient (β = −0.01, 95% CI: −0.09–0.06; *p* = 0.743), and female gender was not significantly associated with acceptance (β = 0.07, 95% CI: −0.03–0.18; *p* = 0.179).

To assess potential multicollinearity among predictors, we examined variance inflation factors (VIFs) and tolerances in regression models; diagnostic results indicated no concerning multicollinearity under conventional thresholds.

Table 7 stratifies the Acceptance Index by prior AI/XR use within preclinical and clinical stages and quantifies effect sizes using Cliff’s δ. Among preclinical students, those with prior AI/XR use (*n* = 49) had substantially higher acceptance (3.1 ± 0.5) than those without prior use (*n* = 71, 2.5 ± 0.5; *p* < 0.001), with a large effect size (Cliff’s δ = 0.60). A similar pattern emerged among clinical students, where prior users (*n* = 33) reported a mean acceptance of 3.4 ± 0.4 compared with 2.9 ± 0.6 among non-users (*n* = 59; *p* < 0.001), again with a large effect size (Cliff’s δ = 0.56).

Table 8 describes the distribution of health literacy (HLS-EU-Q16 [16]) across study stage and prior AI/XR use, as well as mean scores. Overall, most students had sufficient health literacy (136/212, 64.2%), followed by problematic (65/212, 30.7%) and only a small minority with inadequate levels (11/212, 5.2%). Clinical students were somewhat more likely to have sufficient health literacy (71.7%) compared with preclinical students (58.3%), and less likely to have inadequate literacy (1.1% vs. 8.3%). Mean HLS-EU-Q16 scores reflected this pattern, with clinical students scoring higher (14.1 ± 1.5) than preclinical peers (13.3 ± 1.9). When stratified by prior AI/XR use, sufficient health literacy was very common among prior users (70/82, 85.4%) and less frequent among non-users (66/130, 50.8%), who also had higher proportions of problematic and inadequate literacy. The mean health literacy score was correspondingly higher in prior users (14.4 ± 1.3) than non-users (13.2 ± 1.9), suggesting that students with better general health literacy are more likely to have engaged with AI/XR tools.

Table 9 reports a hierarchical linear regression analysis in three models examining how sociodemographic factors, AI/XR attitudes, and health literacy contribute to acceptance of AI/XR-enabled telemedicine. Model 1, including only gender, study stage, and prior AI/XR use, explained 9% of the variance in acceptance (R^2^ = 0.09), with clinical years (β = 0.18, 95% CI: 0.01–0.34; *p* = 0.041) and prior AI/XR use (β = 0.26, 95% CI: 0.11–0.42; *p* = 0.001) emerging as significant predictors. Adding knowledge, perceived accessibility, willingness to pay more, and the HLS-EU-Q16 score [16] in Model 2 substantially improved model fit (R^2^ = 0.42; ΔR^2^ = 0.33, *p* < 0.001): willingness to pay more (β = 0.31, 95% CI: 0.21–0.41; *p* < 0.001), knowledge (β = 0.17, 95% CI: 0.06–0.30; *p* = 0.003), perceived accessibility (β = 0.13, 95% CI: 0.02–0.25; *p* = 0.024), and health literacy (β = 0.07, 95% CI: 0.02–0.13; *p* = 0.009) all made significant contributions, while the effect of prior AI/XR use attenuated but remained significant (β = 0.14; *p* = 0.037). In Model 3, inclusion of the Knowledge × HLS-EU-Q16 interaction term further increased explained variance to 46% (R^2^ = 0.46; ΔR^2^ = 0.04, *p* = 0.012), with the interaction itself significant (β = 0.04, 95% CI: 0.01–0.07; *p* = 0.012), indicating that the positive association between knowledge and acceptance is stronger at higher levels of health literacy. After inclusion of the Knowledge × HLS-EU-Q16 interaction (Model 3), the main effect of knowledge attenuated, consistent with the interpretation that the association of knowledge with acceptance is conditional on health literacy rather than uniform across the sample.

Table 10 summarizes simple-slope analyses examining whether the relationship between knowledge and acceptance differs between low and high health-literacy strata as defined by the HLS-EU-Q16 [16]. Among students with lower health literacy (≤13 points; *n* = 104, mean 12.1 ± 1.3), the slope for knowledge predicting acceptance was modest and did not reach conventional statistical significance (β = 0.12, 95% CI: −0.02–0.26; *p* = 0.089), with the model explaining 18% of the variance (R^2^ = 0.18). In contrast, among students with higher health literacy (≥14 points; *n* = 108, mean 14.7 ± 0.9), knowledge showed a steeper and statistically significant association with acceptance (β = 0.31, 95% CI: 0.12–0.49; *p* = 0.002), and the model explained 29% of the variance (R^2^ = 0.29). The difference between slopes (Δβ = 0.19, 95% CI: 0.04–0.34; *p* = 0.015) confirms a significant moderation effect, indicating that knowledge about AI/XR translates into higher acceptance more strongly among students with sufficient health literacy.

Table 11 presents the results of a k-means cluster analysis (k = 3) that grouped students according to their AI/XR telemedicine attitudes and health literacy, yielding three interpretable clusters. Cluster 1 (“digitally engaged enthusiasts”) comprised 76 students (35.8%) with the highest Acceptance Index (3.6 ± 0.4), high knowledge (3.3 ± 0.5), high willingness to pay (3.3 ± 0.6), and above-average health literacy (HLS-EU-Q16 14.2 ± 1.3). Cluster 2 (“cautious literate pragmatists”) included 89 students (42.0%) with intermediate acceptance (2.8 ± 0.5), moderate knowledge (2.4 ± 0.6), more restrained willingness to pay (2.1 ± 0.7), but relatively good health literacy (13.7 ± 1.7). Cluster 3 (“skeptical low-literacy group”) comprised 47 students (22.2%) with the lowest acceptance (2.3 ± 0.6), lowest knowledge (1.9 ± 0.7), lowest willingness to pay (1.6 ± 0.5), and the lowest health literacy (11.8 ± 1.9). Between-cluster comparisons showed highly significant differences for all variables (*p* < 0.001), with large effect sizes for acceptance (partial η^2^ = 0.32), knowledge (η^2^ = 0.29), and willingness to pay (η^2^ = 0.27), and a moderate effect size for health literacy (η^2^ = 0.20), underscoring the existence of distinct attitudinal and literacy profiles in the student population. Cluster descriptors were chosen to be data-driven and descriptive, reflecting relative profiles across standardized variables rather than value-laden categorizations.

**Figure 1:**
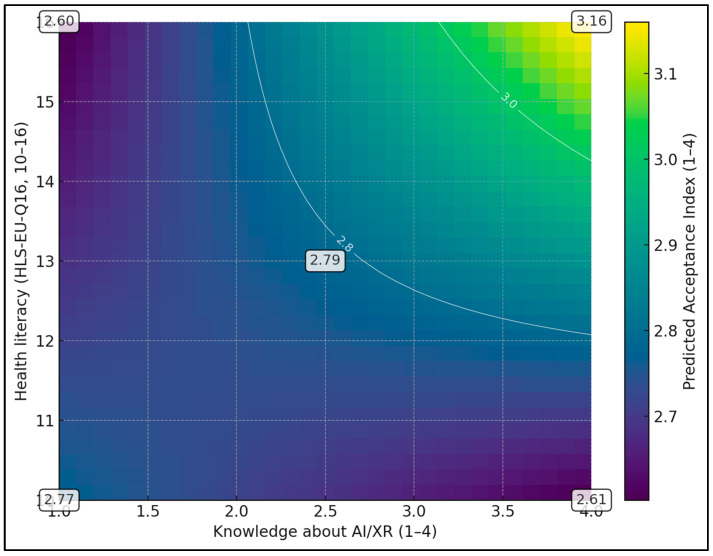
Predicted Acceptance Index by Knowledge and Health Literacy; Lighter regions indicate higher predicted acceptance; the surface illustrates that the knowledge–acceptance slope is steeper at higher health literacy.

Figure 1 displays a heatmap of the predicted Acceptance Index as a joint function of knowledge about AI/XR (*x*-axis, 1–4) and health literacy measured with the HLS-EU-Q16 (*y*-axis, 10–16), with other predictors fixed at typical values (willingness to pay 3.5 and perceived accessibility 3.0). The colour scale ranges from about 2.6 to 3.2 points on the 1–4 Acceptance scale, with lighter colours indicating higher predicted acceptance. At low knowledge (≈1.0) and low health literacy (≈10), predicted acceptance is around 2.6–2.7, whereas at high knowledge (≈4.0) and high literacy (≈16) it increases to ≈3.2, corresponding to a gain of roughly 0.5 points.

**Figure 2:**
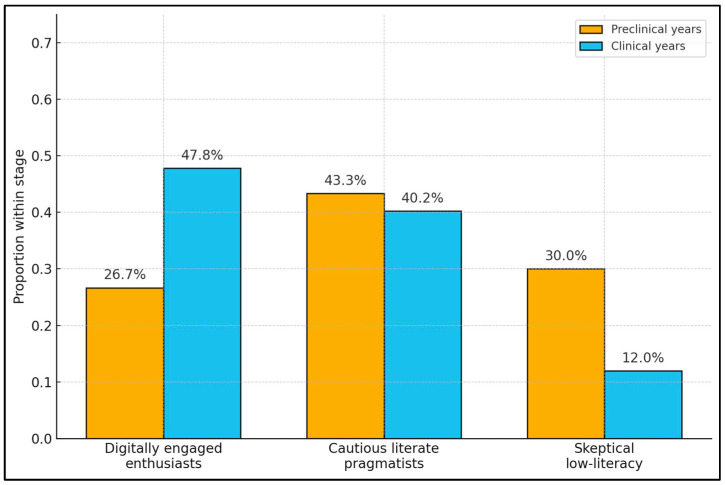
Cluster Composition of AI/XR Attitude Profiles by Study Stage. Bars represent the proportion of students in each cluster within preclinical vs. clinical stages, highlighting heterogeneity in acceptance/knowledge/literacy profiles.

Figure 2 presents the distribution of the three latent clusters identified in the k-means analysis—digitally engaged enthusiasts, cautious literate pragmatists, and skeptical low-literacy students—stratified by study stage. Among preclinical students, enthusiasts comprise 26.7%, pragmatists 43.3%, and skeptics 30.0% of the cohort. In contrast, clinical students show a markedly different profile: enthusiasts almost double in relative weight (47.8%), pragmatists are slightly fewer (40.2%), and skeptics drop to 12.0% (Table 12).

## 4. Discussion

### 4.1. Analysis of Findings

The present survey shows a generally favorable but heterogeneous acceptance of AI/XR-enabled telemedicine among Romanian medical students, with a mean Acceptance Index of 2.9/4 and consistently high willingness to invest extra training time (3.7/4). Acceptance is clearly higher in clinical versus preclinical years (3.1 vs. 2.8) and among prior AI/XR users (3.2 vs. 2.7), suggesting that authentic exposure and clinical responsibility both reinforce positive attitudes. This pattern mirrors cross-sectional data from Iran, where medical students, interns, and residents reported predominantly positive views of telemedicine but highlighted gaps in practical training and infrastructure [17], and from paramedical students whose telehealth attitudes were favorable yet unevenly informed by hands-on experience [18]. Comparable polarization has been documented in Polish university students, where cluster analysis of teleconsultation experiences identified both “enthusiasts” and strongly skeptical groups despite all being digital natives [19]. Acceptance is also likely shaped by institutional and system factors beyond individual attitudes, including the extent of formal curriculum coverage, availability of supervised telemedicine experiences, local digital infrastructure, and the visibility of role-modeling by faculty. Variability in these conditions may partly explain why students at different training stages or with different exposure histories report different acceptance levels

The strong association between AI/XR knowledge and acceptance in our sample (ρ = 0.68) and the independent contribution of prior AI/XR use in multivariable models (β = 0.25) resonate with AI-focused surveys in other regions. In Germany, Austria, and Switzerland, Weidener and Fischer found that over 70% of medical students anticipated a positive impact of AI on medicine, yet only a minority had received formal AI teaching; most explicitly requested integration of AI and AI ethics into the curriculum [20]. A systematic review of healthcare students’ AI attitudes similarly concluded that enthusiasm for AI coexists with limited conceptual understanding and scarce curricular coverage, and recommended embedding AI content within core medical education rather than offering it only as electives [21,22,23]. Our regression results—where knowledge, perceived educational value, and prior AI/XR use jointly explain a large share of variance in acceptance—empirically support these recommendations and suggest that Romanian curricula could leverage even modest AI/XR teaching to convert latent interest into informed, critical acceptance.

Perceived educational value of AI/XR-enabled telemedicine was high in this study (3.1/4 overall, and 3.2/4 among clinical students), and willingness to invest additional training time approached ceiling levels, especially among prior AI/XR users (3.9/4). These findings align with international evidence that digital and simulation-based interventions can enhance knowledge, skills, and engagement when thoughtfully integrated into medical training. A systematic review of digital health training programs for medical students reported improvements in digital competencies and confidence, but also emphasized that most interventions were short, isolated modules rather than longitudinal, curriculum-wide reforms [24]. More recently, a landscape analysis of top-ranked medical schools showed that only a minority explicitly include digital health technologies in their curricula, and almost none reference them in institutional mission statements [25]. Against this backdrop, our data suggest that Romanian students are not resistant to AI/XR in telemedicine; instead, they appear under-served by existing curricular structures and are actively signaling readiness for more structured, longitudinal exposure that links AI/XR tools to concrete learning outcomes.

The integration of a validated health literacy instrument (HLS-EU-Q16) [16] provides additional nuance to the attitudinal picture. Nearly two-thirds of respondents had “sufficient” health literacy (64.2%), and higher health literacy independently predicted greater acceptance in the hierarchical models (β = 0.07 in Model 2) and strengthened the association between knowledge and acceptance, as shown by the significant Knowledge × HLS-EU-Q16 interaction. Similar patterns have been described elsewhere: Ethiopian medical and health-science students with higher eHealth literacy were more likely to use reputable health websites and mobile apps and to perceive online information as useful [22], while Pakistani medical students with higher digital health literacy reported greater confidence in using digital tools and stronger expectations that these tools will be integral to future practice [23]. Taken together, these converging findings suggest that general health and digital health literacy are not merely background traits but active enablers of constructive engagement with AI/XR-supported telemedicine, potentially explaining why privacy concerns in our sample were only weakly related to acceptance despite mid-range concern scores.

Finally, the cluster analysis highlights that students do not form a monolithic group with respect to AI/XR-enabled telemedicine. Our “digitally engaged enthusiasts” combine high acceptance (3.6/4), high knowledge (3.3/4), and high health literacy (14.2/16), while the “skeptical low-literacy” cluster scores lower across all three domains (Acceptance 2.3/4, HLS-EU-Q16 11.8/16). This segmentation resembles the clusters of “enthusiasts,” “indifferent,” and “skeptics/enemies” identified in Polish students’ telemedicine experiences [19] and underscores the value of tailoring educational strategies: advanced, co-creative AI/XR modules for enthusiasts; scaffolded, experience-based introductions for cautious pragmatists; and foundational digital/health-literacy strengthening for skeptical, low-literacy groups. International curriculum analyses show that even globally leading schools have yet to systematically embed digital health technology into mission statements and program structures [25], while systematic reviews of digital health education call for competency-based frameworks that cut across traditional course boundaries [24]. In Romania, where existing work has already documented fragmented telemedicine teaching [9], our results argue for a stratified, literacy-sensitive approach that couples expansion of AI/XR-enabled telemedicine teaching with explicit attention to general and digital health literacy, thereby reducing attitudinal polarization and equipping all future physicians—not just the already-enthusiastic minority—to use these tools responsibly.

One plausible explanation is that health literacy supports effective appraisal of health-related information, including recognizing uncertainty, contextualizing benefits and risks, and calibrating trust in digital outputs. In AI/XR-enabled telemedicine, students with higher health literacy may be better positioned to interpret recommendations as probabilistic support rather than authoritative directives, reducing anxiety and enabling more nuanced judgments about educational value, safety, and privacy. Conversely, lower literacy may amplify cognitive load or uncertainty, weakening the extent to which additional knowledge is converted into confident, critically informed acceptance.

These findings suggest that future Romanian clinicians are broadly ready to work in AI/XR-enhanced telemedicine environments, provided that training is explicit, structured, and aligned with their health literacy level. Higher acceptance in clinical-year students and in those with prior AI/XR experience indicates that supervised exposure during clerkships and simulated teleconsultations could accelerate safe adoption. The strong link between knowledge, perceived educational value, and acceptance supports embedding AI/XR components into formal curricula rather than relying on informal or extracurricular experiences. Cluster patterns—ranging from “digitally engaged enthusiasts” to “skeptical low-literacy” students—highlight the need for differentiated teaching strategies: advanced, hands-on modules for enthusiasts, and more foundational, literacy-sensitive content for skeptical or low-literacy groups. Clinically, better-prepared graduates could improve telemedicine quality, triage efficiency, and patient communication in digitally enabled services, while remaining attentive to privacy and ethical issues.

These findings indicate a strong association between knowledge and acceptance; however, given the cross-sectional design, we cannot infer directionality, and it is also possible that students with higher acceptance seek more exposure or information about AI/XR.

Practical curriculum implications include: (i) early, structured AI fundamentals (limitations, bias, uncertainty, and evaluation), (ii) AI ethics and data governance modules aligned with privacy-by-design principles, (iii) hands-on XR-supported teleconsultation simulations during clinical years (e.g., supervised OSCE-style telemedicine stations), and (iv) literacy-scaffolded learning pathways that explicitly teach students how to appraise digital outputs and communicate uncertainty to patients.

### 4.2. Study Limitations

This study has important limitations. First, the cross-sectional design precludes causal inference and does not capture how acceptance evolves with training or exposure. Second, the sample was drawn from a single institution using convenience recruitment, which limits generalizability and introduces potential self-selection bias (students more interested in digital health may have been more likely to respond). Third, measures were self-reported, raising the possibility of common method bias and socially desirable responding, particularly for attitudes toward emerging technologies. Fourth, while internal consistency of the Acceptance Index was acceptable, the measure remains study-specific and requires external psychometric validation. Finally, we did not assess objective competencies (observed telemedicine performance, communication quality, or error rates) or conduct longitudinal follow-up. Future studies should include multi-institutional sampling, longitudinal designs, and ideally interventional evaluations of AI/XR teaching that measure both attitudes and objectively assessed skills.

## 5. Conclusions

In this single-centre survey of Romanian medical students, acceptance of AI/XR-enabled telemedicine was generally high and strongly driven by domain-specific knowledge, perceived educational value, prior AI/XR exposure, and clinical study stage, while privacy concerns played a limited role. Health literacy emerged as a key contextual factor that not only associated with higher acceptance but also amplified the positive impact of knowledge on attitudes. Distinct attitudinal and literacy clusters underscore that the student body is not homogeneous and that “one-size-fits-all” digital education strategies are unlikely to be optimal. Together, these results support early, scaffolded integration of AI and XR into telemedicine teaching, combined with targeted support for students with lower health literacy or more skeptical profiles. Such an approach may help prepare a future medical workforce that can use AI/XR tools critically, ethically, and effectively in increasingly digital healthcare systems. Future educational interventions could include early AI/XR literacy modules in preclinical years and supervised XR-supported teleconsultation simulations in clinical years, with explicit training in ethics, privacy, and uncertainty communication. Further research should test these approaches using multi-institutional longitudinal or interventional designs that measure both attitudes and objective telemedicine competencies

## Figures and Tables

**Table 1:** Acceptance Index.

Measure/Item	Wording	Scale	Direction
Acceptance Index (A1)	Trust in AI-assisted recommendations during a teleconsultation (educational setting).	1–4	Higher = higher acceptance
Acceptance Index (A2)	AI/XR would improve the quality of telemedicine encounters for learning.	1–4	Higher = higher acceptance
Acceptance Index (A3)	Willingness to participate in AI/XR-enabled teleconsultations as part of training.	1–4	Higher = higher acceptance
Knowledge (K1)	Self-rated knowledge about AI/XR applications in healthcare and education.	1–4	Higher = more informed
Perceived educational value (V1)	AI/XR-enabled telemedicine would add educational value to training.	1–4	Higher = greater perceived value
Privacy concern (P1)	Concern about privacy/data protection in AI-assisted teleconsultations.	1–4	Higher = more concern (not reverse-scored)
Willingness to invest time (WTT1)	Willingness to invest additional personal time to learn AI/XR tools.	1–4	Higher = more willing
Perceived accessibility (ACC1)	AI/XR-enabled telemedicine would be accessible/feasible for students in this setting.	1–4	Higher = more accessible
Willingness to pay more (WTP1)	Willingness to pay more/accept added cost for AI/XR-enhanced telemedicine training.	1–4	Higher = more willing

**Table 2:** Demographic and exposure characteristics by study stage.

Variable	Total (*n* = 212)	Preclinical (*n* = 120)	Clinical (*n* = 92)	*p*-Value
Age, years, mean ± SD	22.5 ± 1.9	21.2 ± 1.2	24.1 ± 1.3	<0.001
Female, *n* (%)	140 (66.0%)	82 (68.3%)	58 (63.0%)	0.509
Prior telemedicine use, *n* (%)	120 (56.6%)	60 (50.0%)	60 (65.2%)	0.038
Prior AI/XR use, *n* (%)	82 (38.7%)	49 (40.8%)	33 (35.9%)	0.553

Abbreviations: SD, standard deviation; AI, artificial intelligence; XR, extended reality.

**Table 3:** AI/XR-related attitudinal scores by study stage.

Variable	Total Mean ± SD	Preclinical Mean ± SD	Clinical Mean ± SD	*p*-Value
Knowledge about AI/XR (1–4)	2.4 ± 0.8	2.3 ± 0.8	2.7 ± 0.8	<0.001
Perceived educational value (1–4)	3.1 ± 0.6	3.0 ± 0.6	3.2 ± 0.6	0.023
Privacy concern (1–4; higher = more concern)	2.5 ± 0.7	2.4 ± 0.6	2.6 ± 0.7	0.094
Acceptance Index (1–4)	2.9 ± 0.6	2.8 ± 0.5	3.1 ± 0.6	<0.001
Willingness to invest extra training time (1–4)	3.7 ± 0.5	3.7 ± 0.5	3.8 ± 0.4	0.013

Abbreviations: AI, artificial intelligence; XR, extended reality; SD, standard deviation.

**Table 4:** AI/XR-related scores by prior AI/XR use (all years combined).

Variable	Prior AI/XR Use: Yes (*n* = 82)	Prior AI/XR Use: No (*n* = 130)	*p*-Value
Acceptance Index (1–4)	3.2 ± 0.5	2.7 ± 0.6	<0.001
Knowledge about AI/XR (1–4)	2.9 ± 0.7	2.1 ± 0.8	<0.001
Perceived educational value (1–4)	3.3 ± 0.6	3.0 ± 0.6	<0.001
Privacy concern (1–4)	2.6 ± 0.7	2.4 ± 0.7	0.084
Willingness to invest training time (1–4)	3.9 ± 0.3	3.6 ± 0.6	<0.001

Abbreviations: AI, artificial intelligence; XR, extended reality.

**Table 5:** Spearman correlations between key attitudinal variables.

Variable	Knowledge	Acceptance	Value	Privacy	WTT
Knowledge	—	0.68 (<0.001)	0.49 (<0.001)	0.05 (0.463)	0.42 (<0.001)
Acceptance	0.68 (<0.001)	—	0.60 (<0.001)	0.05 (0.498)	0.36 (<0.001)
Value	0.49 (<0.001)	0.60 (<0.001)	—	0.03 (0.621)	0.32 (<0.001)
Privacy	0.05 (0.463)	0.05 (0.498)	0.03 (0.621)	—	0.03 (0.651)
WTT (time)	0.42 (<0.001)	0.36 (<0.001)	0.32 (<0.001)	0.03 (0.651)	—

Abbreviations: WTT, willingness to invest extra training time. Correlation analyses summarize bivariate associations among attitudes and acceptance. Regression models then estimate independent predictors of acceptance while adjusting for training stage, prior exposure, and co-occurring attitudes, thereby addressing overlap among predictors.

**Table 6:** Multiple linear regression predicting Acceptance Index.

Predictor	β (Unstandardized)	95% CI	*p*-Value
Constant	1.04	0.72 to 1.36	<0.001
Knowledge (1–4)	0.28	0.20 to 0.36	<0.001
Value (1–4)	0.32	0.23 to 0.41	<0.001
Privacy (1–4)	−0.01	−0.09 to 0.06	0.743
Prior AI/XR use (yes vs. no)	0.25	0.13 to 0.37	<0.001
Stage (clinical vs. preclinical)	0.18	0.07 to 0.29	0.001
Gender (female vs. male)	0.07	−0.03 to 0.18	0.179

Dependent variable: Acceptance Index (1–4). R^2^ = 0.61. Abbreviations: AI, artificial intelligence; XR, extended reality; β, unstandardized regression coefficient; CI, confidence interval; R^2^, coefficient of determination.

**Table 7:** Acceptance by prior AI/XR use within preclinical and clinical stages.

Stage	Prior AI/XR Use	*n*	Acceptance Mean ± SD	*p*-Value (Within Stage)	Cliff’s δ
Preclinical	Yes	49	3.1 ± 0.5	<0.001	0.6
Preclinical	No	71	2.5 ± 0.5	—	—
Clinical	Yes	33	3.4 ± 0.4	<0.001	0.56
Clinical	No	59	2.9 ± 0.6	—	—

Abbreviations: AI, artificial intelligence; XR, extended reality; SD, standard deviation; δ, Cliff’s delta (effect-size measure).

**Table 8:** Health literacy (HLS-EU-Q16) distribution by study stage and prior AI/XR use.

HLS-EU-Q16 Level	Overall (*n*= 212) *n* (%)	Preclinical Years (*n* = 120) *n* (%)	Clinical Years (*n* = 92) *n* (%)	Prior AI/XR Use: Yes (*n* = 82) *n* (%)	Prior AI/XR Use: No (*n* = 130) *n* (%)
Inadequate (0–8)	11 (5.2%)	10 (8.3%)	1 (1.1%)	2 (2.4%)	9 (6.9%)
Problematic (9–12)	65 (30.7%)	40 (33.3%)	25 (27.2%)	10 (12.2%)	55 (42.3%)
Sufficient (13–16)	136 (64.2%)	70 (58.3%)	66 (71.7%)	70 (85.4%)	66 (50.8%)
Mean HLS-EU-Q16 score (0–16)	13.6 ± 1.8	13.3 ± 1.9	14.1 ± 1.5	14.4 ± 1.3	13.2 ± 1.9

Abbreviations: HLS-EU-Q16, European Health Literacy Survey Questionnaire, 16-item short form; AI, artificial intelligence; XR, extended reality.

**Table 9:** Hierarchical linear regression predicting Acceptance Index (1–4) from sociodemographic factors, AI/XR attitudes, and health literacy.

Predictor	Model 1 β (95% CI), *p*	Model 2 β (95% CI), *p*	Model 3 β (95% CI), *p*
Intercept	2.41 (2.09–2.72), *p* < 0.001	1.39 (0.88–1.90), *p* < 0.001	1.27 (0.76–1.81), *p* < 0.001
Female (vs. male)	0.11 (−0.03–0.25), *p* = 0.129	0.07 (−0.05–0.20), *p* = 0.249	0.06 (−0.06–0.19), *p* = 0.302
Clinical years (vs. preclinical)	0.18 (0.01–0.34), *p* = 0.041	0.09 (−0.04–0.23), *p* = 0.172	0.08 (−0.06–0.22), *p* = 0.259
Prior AI/XR use (yes vs. no)	0.26 (0.11–0.42), *p* = 0.001	0.14 (0.01–0.27), *p* = 0.037	0.12 (−0.01–0.25), *p* = 0.068
Knowledge (1–4)	–	0.17 (0.06–0.30), *p* = 0.003	0.09 (−0.03–0.22), *p* = 0.133
Perceived Accessibility (1–4)	–	0.13 (0.02–0.25), *p* = 0.024	0.11 (0.01–0.23), *p* = 0.038
Willingness to Pay more (1–4)	–	0.31 (0.21–0.41), *p* < 0.001	0.28 (0.18–0.39), *p* < 0.001
HLS-EU-Q16 score (0–16)	–	0.07 (0.02–0.13), *p* = 0.009	0.05 (−0.01–0.11), *p* = 0.096
Knowledge × HLS-EU-Q16 (centered)	–	–	0.04 (0.01–0.07), *p* = 0.012
Model R^2^	0.09	0.42	0.46
ΔR^2^ vs. previous model	–	+0.33, *p* < 0.001	+0.04, *p* = 0.012

Abbreviations: HLS-EU-Q16, European Health Literacy Survey Questionnaire, 16-item short form; β, unstandardized regression coefficient; CI, confidence interval; R^2^, coefficient of determination; ΔR^2^, change in R^2^; WTP, willingness to pay more.

**Table 10:** Simple slopes of Knowledge → Acceptance Index at low vs. high health literacy.

HLS-EU-Q16 Stratum	*n*	Mean HLS-EU-Q16 (±SD)	β for Knowledge (1–4) → Acceptance (1–4)	95% CI for β	*p*-Value	Model R^2^
Low HL (≤13)	104	12.1 ± 1.3	0.12	−0.02 to 0.26	0.089	0.18
High HL (≥14)	108	14.7 ± 0.9	0.31	0.12 to 0.49	0.002	0.29
Difference in slopes	–	–	Δβ = 0.19	0.04 to 0.34	0.015	–

Abbreviations: HLS-EU-Q16, European Health Literacy Survey Questionnaire, 16-item short form; HL, health literacy; β, unstandardized regression coefficient; CI, confidence interval; R^2^, coefficient of determination.

**Table 11:** Cluster analysis of AI/XR telemedicine attitudes and health literacy (k = 3).

Cluster Label (Interpretive)	*n* (%)	Acceptance Index (Mean ± SD)	Knowledge (1–4) (Mean ± SD)	WTP (1–4) (Mean ± SD)	HLS-EU-Q16 (0–16) (Mean ± SD)
Cluster 1: High acceptance	76 (35.8%)	3.6 ± 0.4	3.3 ± 0.5	3.3 ± 0.6	14.2 ± 1.3
Cluster 2: Moderate acceptance	89 (42.0%)	2.8 ± 0.5	2.4 ± 0.6	2.1 ± 0.7	13.7 ± 1.7
Cluster 3: Low acceptance	47 (22.2%)	2.3 ± 0.6	1.9 ± 0.7	1.6 ± 0.5	11.8 ± 1.9

Between-cluster comparisons (ANOVA/Kruskal–Wallis): Acceptance Index: F(2, 209) = 49.3, *p* < 0.001, partial η^2^ = 0.32; Knowledge: F(2, 209) = 41.7, *p* < 0.001, partial η^2^ = 0.29; WTP: F(2, 209) = 38.5, *p* < 0.001, partial η^2^ = 0.27; HLS-EU-Q16: F(2, 209) = 26.8, *p* < 0.001, partial η^2^ = 0.20. Abbreviations: WTP, willingness to pay; HLS-EU-Q16, European Health Literacy Survey Questionnaire, 16-item short form; SD, standard deviation; ANOVA, analysis of variance; η^2^, eta-squared (effect-size index); k-means, k-means clustering algorithm.

**Table 12:** VIF/Tolerance Collinearity Diagnostics.

Predictor	Approx. VIF	Approx. Tolerance (1/VIF)
Knowledge	3.3	0.3
Perceived Value	2.1	0.48
Privacy Concern	1.4	0.73
Prior AI/XR Use	1.5	0.68
Clinical Stage	1.3	0.77
Gender	1.2	0.85

## Data Availability

The data presented in this study are available on request from the corresponding author.
